# Transdifferentiation of Human Dental Pulp Stem Cells Into Oligoprogenitor Cells

**DOI:** 10.18869/nirp.bcn.8.5.387

**Published:** 2017

**Authors:** Ardeshir Moayeri, Maryam Nazm Bojnordi, Sara Haratizadeh, Amir Esmaeilnejad-Moghadam, Rafieh Alizadeh, Hatef Ghasemi Hamidabadi

**Affiliations:** 1. Department of Anatomy, Faculty of Medicine, Ilam University of Medical Sciences, Ilam, Iran.; 2. Department of Anatomy & Cell Biology, Faculty of Medicine, Mazandaran University of Medical Sciences, Sari, Iran.; 3. Molecular & Cell Biology Research Center, Department of Anatomy & Cell Biology, Faculty of Medicine, Mazandaran University of Medical Sciences, Sari, Iran.; 4. ENT and Head & Neck Research Center and Department, Hazrat Rasoul Akram Hospital, Iran University of Medical Sciences (IUMS), Tehran, Iran.; 5. Immunogenetic Research Center, Department of Anatomy and Cell Biology, Faculty of Medicine, Mazandaran University of Medical Sciences, Sari, Iran.

**Keywords:** Dental pulp stem cells, Regenerative medicine, Oligoprogenitor cells, Mesenchymal stem cells

## Abstract

**Introduction:**

The nerve fibers in central nervous system are surrounded by myelin sheet which is formed by oligodendrocytes. Cell therapy based on oligodendrocytes and their precursors transplantation can hold a promising alternative treatment for myelin sheet repair in demyelinating diseases.

**Methods:**

Human Dental Pulp Stem Cells (hDPSCs) are noninvasive, autologous and easy available source with multipotency characteristics, so they are in focus of interest in regenerative medicine. In the present study, hDPSCs were differentiated into oligoprogenitor using glial induction media, containing Retinoic Acid (RA), basic Fibroblast Growth Factor (bFGF), Platelet-Derived Growth Factor (PDGF), N2 and B27. The differentiated Oligoprogenitor Cells (OPCs) were evaluated for *nestin, Olig2, NG2* and *O4* using immunocytochemistry. Also, the expression of nestin, *Olig2* and *PDGFR-α* gens (neuroprogenitor and oligoprogenitor markers) were investigated via RT-PCR technique.

**Results:**

The results indicate that glial differentiation medium induces the generation of oligoprogenitor cells as revealed via exhibition of specific glial markers, including *Olig2*, NG2 and O4. The expersion of nestin gene (neuroprogenitor marker) and *Olig2* and PDGFR-α genes (oligoprogentor markers) were detected in treated hDPSCs at the end of the induction stage.

**Conclusion:**

hDPSCs can be induced to transdifferentiate into oligoprogenitor cells and respond to the routinely applied regents for glial differentiation of mesanchymal stem cells. These data suggest the hDPSCs as a valuable source for cell therapy in neurodegenerative diseases.

## 1. Introduction

Myelin sheet disruption or degeneration causes severe neurological dysfunction as well as disability in patients with demyelinating diseases such as Multiple Sclerosis (MS) (Delilovic-Vranic, 2005). Because oligodendrocyte forms the myelin sheet, oligodendrocyte-based cell therapy is suggested as a promising alternative therapy for myelin repair of demyelinated nerves (Baumann & Pham-Dinh, 2001; Blakemore & Franklin, 2007). The cell type candidate for transplantation is very important because of ethical issuses, tumorigenic activity and possible rejection of transplanted cells (Patani & Chandran, 2012; Nazm bojnordi, Movahedin, Tiraihi, Javan, & Ghasemi Hamidabadi, 2013, Schippling, Heesen, Zander, & Martin, 2008). Mesenchymal Stem Cells (MSCs) are considered as a feasible source that overcome these limitations (Haratizadeh, Nazm Bojnordi, Niapour, Bakhtiari, Ghasemi Hamidabadi, 2016; Nazm bojnordi. Ghasemi, & Akbari, 2015). They are multipotent stem cells with the neurogenic potential that make them good candidates for different types of nervous tissues (Chopp & Li, 2002; Pluchino & Martino, 2005).

Human dental pulp stem cells are multipotent stem cells that can be considered as a new noninvasive autologous source for MSCs (Ghasemi et al., 2017; Nagatomo et al., 2006; Sloan & Smith, 2007). They show neural characteristics like neurons and can be easily collected from dental tissues (Huang, Chen, Lin, Shieh, & Chan 2008; Gronthos, Mankani, Brahim, Robey, & Shi, 2000). These properties nominate hDPSCs as an appropriate cell source for neuroregenerative medicine. Up to date, the multipotency potential of hDPSCs to generate different linages such as osteogenic, adipogenic as well as neurogenic lines have been investigated (Sloan & Smith, 2007; Gronthos S et al., 2002). Although differentiation of hDPSCs into neuron cell type has been reported (Chang, Chang, Tsai, Chang, & Lin, 2014; Chun, Soker, Jang, Kwon, & Yoo, 2016), the in-vitro oligodendrogenesis potential of these cells and assessment of mature specific markers of differentiated cells have been overlooked. In this regard, previous research studies showed that transplantation of differentiated cells has more effective than engraftment of undifferentiated stem cells. To this end, we planned to access the oligoprogenitor cells that are more restricted to the glial lineage.

Here, we differentiated hDPSCs to oligodendrocyte progenitor cells under appropriate conditions in vitro and evaluate the generation of oligoprogenitor cells using the expression of nestin (neuroprogenitor marker) and specific glial markers, i.e. *Olig2*, *NG2, O4* and *PDGFR-α* genes by immunocytochemistry and RT-PCR techniques.

The expression of these glial specific markers has not been evaluated in previous reports. Glial differentiation potential of MSCs has been demonstrated previously by using different induction protocols and various chemical inducers. According to standard protocol for glial differentiation of MSCs, the aim of this research was to improve the induction technique for in vitro differentiation of hDPSCs into oligoprogenitor cells using retinoic acid and growth factors such as basic fibroblast growth factor, platelet-derived growth factor, N2 and B27. The finding of this study suggest the hDPSCs as an alternative stem cell source usable in oligodendrogenesis in vitro for treatment of demyelinating diseases.

## 2. Methods

### 2. 1. Extraction and culture of hDPSCs

Human dental pulp tissue were collected from healthy third molar teeth of clients referring to a dental clinic affiliated to Mazandaran University of Medical Sciences, Sari, Iran. Pulp tissue were minced and digested using mechanical and enzymatic digestion with trypsin 0.25% (Gibco, USA) enzyme. After centrifuge of tissue pieces, the supernatant was removed and cultured in medium then incubated in DMEM/F12 supplemented with 15% FBS, streptomycin /penicillin, and L-glutamine. Growth and morphological features of cells were monitored every 2–3 days via inverted microscope.

### 2.2. Multilineage differentiation of hDPSCs

The multipotency of hDPSCs was investigated by their differentiation into adipocyte and osteoblast according to adipogenic and osteogenic differentiation protocols. Alizarin Red and Oil Red O staining were respectively used for evaluation of osteogenic and adipogenic activity of treated cells.

### 2.3. Flow cytometry

The immunophenotypic detection of mesenchymal stem cell markers i.e. CD90, CD44, CD105 and hematopoietic stem cell markers, i.e. CD34 and CD45 was performed by flow cytometry technique.

### 2.4. Differentiation of hDPSCs

We used preinduction and induction according to glial differentiation protocol for mesenchymal stem cells (Sanchez-Ramos et al., 2000). hDPSc at fourth passage were preinduced in the presence of DMEM-F12 medium, containing FBS 5% and retinoic acid (Sigma Aldrich), 1M for 4 days. In the induction stage, the cells were incubated in DMEM/F12 medium in the presence 5 ng/mL platelet-derived growth factor (Sigma Aldrich) and 10 ng/mL basic fibroblast growth factor (Sigma Aldrich) for 8 days.

### 2.5. MTT Test

Viability of isolated cells was carried out by Methyl Thiazolyl Tetrazolium (MTT) in day 4 and 12 (preinduction and induction stages). First of all, 4×104 cells were transferred to all 6-well plate sinks. Then the cells were cultured in the incubator under standard conditions of temperature and humidity. After incubation, the medium was removed and replaced with 50 μL of Dimethyl Sulfoxide (DMSO), then placed on a shaker for 5–10 min to agitate and dissolve the formazan crystals. Absorbance at 570 nm was measured in a Cytofluor 4000 plate reader (PerSeptive Biosystems, Framingham, Massachusetts, USA). All experiments were performed in three replicate wells.

### 2.6. Immunocytochemistry analysis

At the end of induction stage, the cells were harvested for evaluation of glial specific markers i.e. *Olig2, NG2* and *O4* to confirm glial differentiation of hDPSc. Also, nestin marker was examined at the end of preinduction stage.

Cells in each group were fixed in 4% paraformaldehyde (pH=7.4) for 30 min at Room Temperature (RT). Fixed cells were permeabilized with 0.2% Triton X-100 for 10 min followed by three washes with PBS then were blocked by 10% goat serum for 30 min. Primary antibodies, including mouse *anti-Olig2* monoclonal antibody (abcam) (1:200), mouse *anti-NG2* monoclonal antibody (abcam) (1:200), mouse *anti-O4* monoclonal antibody (abcam) (1:300) that are specific markers for OPc were applied. The following day, the cells were washed twice with PBS and incubated with the appropriate secondary antibody; Fluorescein Isothiocyanate (FITC) secondary antibody IgG (1:1000) for 1 hour at room temperature. After washing with PBS, cells were mounted with 4,6-diamidino-2-phenylindole (DAPI)/PBS (1:1000) for 1 min and images were captured with an Olympus phase.

### 2.7. RT-PCR

At the end of induction stage, hDPSCs were evaluated for the expression of nestin, *Olig2* and *PDGFR-α* genes. RNX-Plus Kit (Fermentas) was used for RNA extraction followed conversion of extracted RNA to cDNA by the cDNA Synthesis Kit (Ferments). PCR reaction was done by adding 50 ng of cDNA for 35 cycles following denaturation for 45 seconds at 95°C, annealing for 45 seconds at 58°C, and elongation for 30 seconds at 72°C. Primer sequences of nestin gene (neuroprogenitor marker) evaluated using the *5′-GGAGTCCTGGATTTCCTTCC-3′* and *5′-GCCCTGACCACTCCAGTTT-3*. Primer sequences of *Olig2* gene (oligoprogenitor marker) evaluated using the *5′-GCTGCGTCTCAAGATCAAC-3′* and *5′-AGTCGCTTCATCTCCTCCA-3′* and primer sequence of *PDGFR-α* gene (marker for oligoprogenitor) evaluated using the *5′-GTGGGACATTCATTGCGGA-3′* and *5′-AAGCTGGCAGAGGATTAGG-3′*. Primer sequences of β-actin gene (Internal control) evaluated using *5′-GACTTCGAGCAAGAGATGG-3′* and *5′-GACAGCACTGTGTTGGCGTA-3′*.

### 2.8. Statistical analysis

The statistical analysis was performed with SPSS 13.0 software applying 1-way Analysis of Variance (ANOVA) followed by Tukey post hoc test. P less than 0.05 was considered significant. Each point represents the average of three separate experiments

## 3. Results

### 3.1. Characterization of human dental pulp stem cells

**Figure 1.**
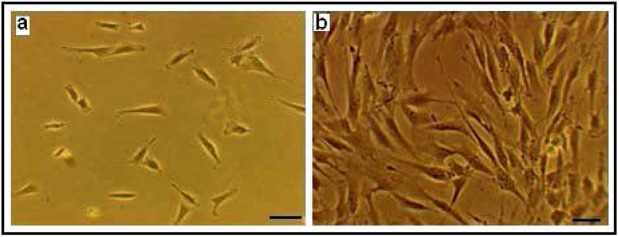
Human Dental pulp stem cells culture (×100). (a) Primary culture after 12h culture; (b) After 4th passage culture.

**Figure 2.**
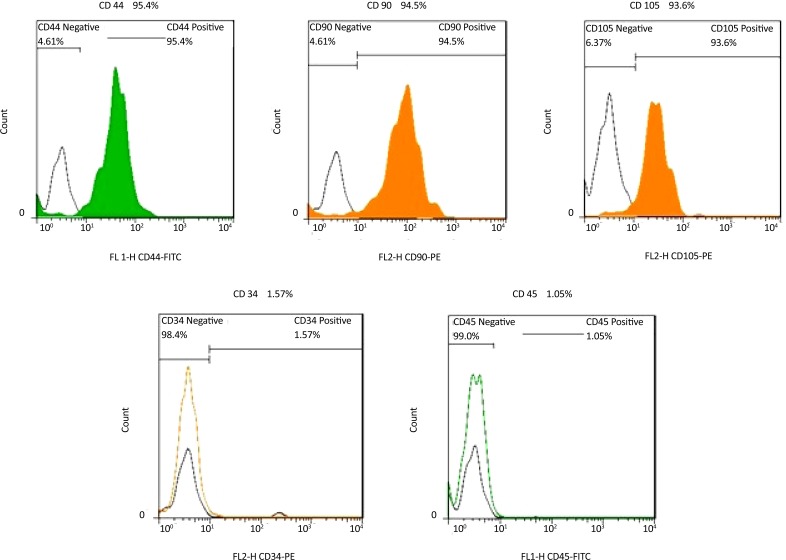
Flow cytometric analysis of hDPSCs. Cells after fourth passage were strongly immunopositive to specific surface markers of mesenchymal stem cells; CD44, CD90, CD105 but didn’t express CD34 and CD45 which are specific markers to hematopoietic stem cells.

**Figure 3.**
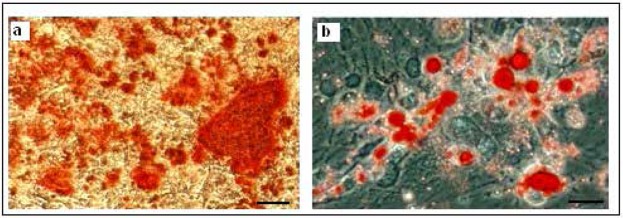
Possibility of Multilineage differentiation of Dental pulp stem cells (×100). (a) Alizarin Red staining that shows osteogenic differentiation of hDPSC. (b) Oil Red O staining for detection of lipid droplets which confirms adipogenic differentiation.

The isolated hDPSCs had elongated shaped at the onset of culture. They had the ability of colony formation, with a high proliferation and adherence activity that filled the flask bottom and then subcultured. Subcultured cells exhibited flattened and fibroblastic morphology. These phenotype confirm the mesenchymal characteristics of isolated hDPSCs (Figure 1). The isolated stem cells were positive for the mesenchymal antigens CD 44, CD 90, CD 105 using flow cytometry. Also they had a negative tendency to CD34, CD45 i.e. hematopoietic markers (Figure 2). Multilineage differentiation of hDPSCs was investigated their MSC characteristic. Osteogenic differentiation was confirmed by production of calcium deposits via Alizarin Red staining (Figure 3a). Adipogenic differentiation was confirmed by Oil Red O staining of lipid droplet (Figure 3b).

### 3.2. Differentiation of hDPSCs into OPCs

**Figure 4.**
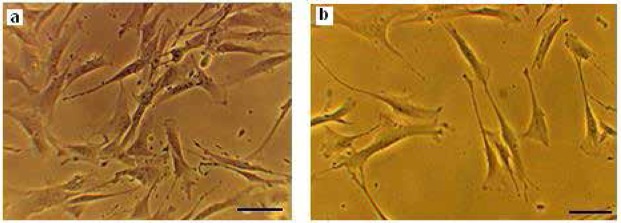
Differentiation human Dental pulp stem cells into oligoprogenitor cells. Neuroprogenitor cells after preinduction phase (a). Further cultivation, lead to generation of OPC at the end of induction stage (c). Scale bars 10 μm.

**Figure 5.**
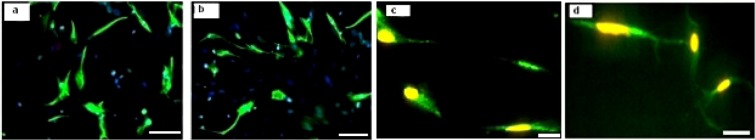
Immunofluorescence staining for human Dental pulp stem cells differentiation into oligoprogenitor cells. Immunostained cells with (a) Anti-Nestin, (b) Anti-olig2, (c) Anti-NG2 and (d) Anti-O4 antibodies. Scale bars 10 μm.

Glial differentiation of hDPSCs was associated with the generation of neuroprogenitor cells at the first step of differentiation protecole. After exposure to RA, DPSCs acquired a phenotype resembling neuroprogenitor cells. Neuroprogenitor cells differentiated into OPCs at the end of the induction stage. Morphological changes appeared during differentiation process i.e. spindle like hDPSCs are converted to branched shape OPCs (Figure 4). The results of immunoflourcent staining showed that the preinduced cells expressed nestin that is considered as a neuroepithelial marker. The mean percentages of immunoreactive cells to this marker was 69.3%±1.54% (Figure 5a). Also, the immunostaining of induced cells indicated the expression of specific glial markers such as *Olig2*, *NG2* and *O4* at the end of differentiation protocol (Figure 5 b, c, d). The mean percentages of immunoreactive cells to mentioned markers were 47.5%±5.19%, 42.7%±3.49% and 39.63%±1.93%, respectively.

### 3.3. Survival rate of hDPSCs during differentiation protocol

**Figure 6.**
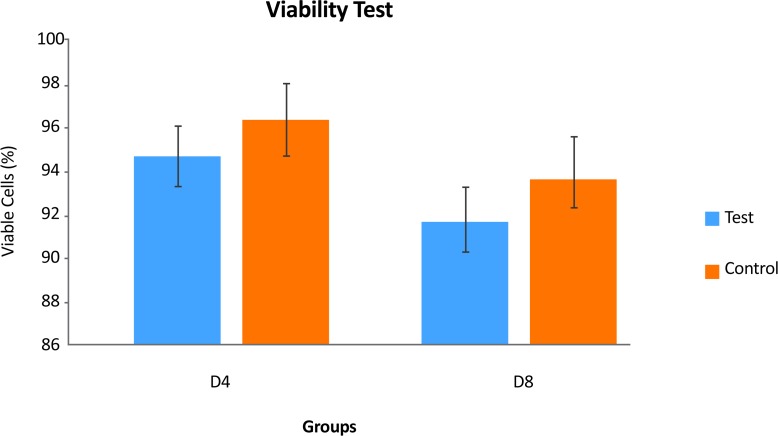
The comparison between the mean viability rates at pre-induction (a) and induction (b) stages or in days (D) D4 (a) and D12 (b) (P<0.05). a: significant decrease with test group in D4.

MTT results showed that the viability of the cells at preinduction stage in the test and control groups, were respectively, 94.71%±0.26% and 96.13%±0.58%. The percentages of viable cells in the treated group at the induction stage was lower than in the control group. A significant decrease was seen in cell proliferation rate following induction stage (P<0.05) (Figure 6).

### 3.4. RT-PCR

**Figure 7.**
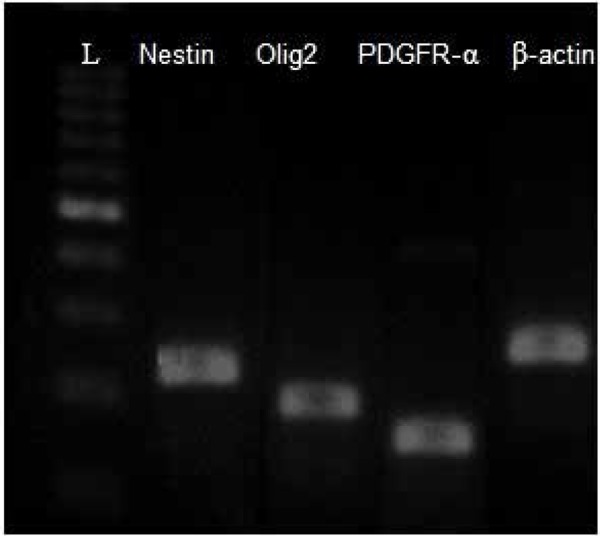
RT-PCR detection of Nestin (220 bp), *Olig2* (192), PDGFR-α (124 bp) and β-actin (237 bp) in the differentiated hDPSCs group. The expersion of these gene were detectable in induced cells. L=DNA ladder.

RT-PCR results proved the expression of nestin gene (neuroprogenitor marker) and *Olig2* and *PDGFR-α* genes (oligoprogentor markers) in treated hDPSCs at the end of the induction stage, while no gene expression was detected in untreated hDPSCs (Figure 7).

## 4. Discussion

The neurogenic activity of mesenchymal stem cells in culturing with the presence of neural inducers have been evaluated in previous literature (Black & Woodbury, 2001; Jiang et al., 2010). Also, the neural crest origin of hDPSCs proposes them as an alternative source for neuroglial cell population for therapeutic strategies in neuroregenerative medicine (Yalvac et al., 2009; Gervois et al., 2015). But a few studies investigated the in vitro differentiation potential of hDPSCs to oligoprogenitor cells.

So we proposed the culturing of hDPSCs in the presence of glial inducers, e.g. bFGF, PDGF, N2 and B27 similar to standard glial differentiation protocols for MSC or BMSCs which can generate oligoprogenitor cells. They can be candidates for the treatment of demyelinating diseases (Liu et al., 2007). The multilineage differentiation of hDPSCs into adipocytes and osteocyte cells was confirmed with specific staining respectively; Oil Red O and Alizarin Red staining. This result is in agreement with previous reports about the multipotency of hDPSCs as well as MSC.

Our findings showed that glial generation of hDPSCs is associated with the generation of neuroprogenitor cells at the first step of differentiation. After exposure to inducers, hDPSCs were converted from proliferation phase to differentiation stage and acquired a phenotype resembling neuroprogenitor cells. Furthermore, the pre-induced cells were immunopositive to nestin while no expression of this marker was detectable in untreated hDPSCs. These data confirm the previous reports about glial generation associated with nestin that utilized as neuroprogenitor markers during neurogenesis (Wislet-Gendebien, 2005; Gu et al., 2015; Bojnordi et al., 2017).

The cells were treated with PDGFF, bFGF, N2 and B27 following preinduction stage. They completed glial differentiation and were immunopositive to specific glial markers. The fluorescence staining for *Olig2*, *NG2* and *O4* indicates the immune positive reaction of OPCs to these glial markers. The percentages of *Olig2*, *NG2* and *O4* positive cells were significantly higher in this experiment treated group compared to hDPSCs cultured in control media. Addition of inducers such as PDGFF, bFGF, N2 and B27 can promote differentiation of hDPSCs into oligodendrocyte progenitors.

Furthermore, RT-PCR results proved the expression of nestin, *Olig2* and *PDGFR-α* genes (oligoprogentor markers) in treated hDPSCs at the end phase of the induction protocol. Several reports have demonstrated the active role of these factors in oligodendrogenesis as well as myelination. The expression of *Olig2* after induction phase is in agreement with the findings of Liu et al., 2007 and Copray et al., 2006. This helix-loop transcription factor has important role in glial fate map in oligodendrogenesis process. Similar results were reported that showed the generation of mesenchymal stem cells into oligodendrocytes is accompanied with overexpression of specific glial genes (Ni et al., 2010; Sanchez-Ramos et al., 2000). The results of MTT test showed a decrease in cell proliferation rate following induction stages that confirms the differentiation of hDPSCs following exposure to glial induction media instead of proliferation activity.

hDPSCs are multipotent cells with high proliferation rate as well as plasticity for differentiation to various cell types. In total, hDPSCs can transdifferentiate to oligoprogenitor cells after two steps differentiation protocol. The glial inducers include RA, PDGFF, bFGF and EGF as well as the ommition of serum, totally alter the cells microenvironment and induce differentiation of hDPSCs to oligoprogenitor cells.

Our results proved the differentiation capacity of hDPSCs to oligoprogenitor cells that exhibit special glial morphology and express the special oligoprogenitor markers investigated by immunocytochemistry technique. It concludes that differentiation of hDPSCs into oligoprogenitor cells depends on addition of glial inducers which are currently used for MSC or BMSC. This finding supports the therapeutic use of hDPSCs as an alternative source for generation of glial cells for the clinical repair of demyelinating diseases. However, more studies are necessary to be done to evaluate the complete differentiation of hDPSCs to fully functional oligodendrocytes.
